# Is Posterior Cruciate Ligament Needle Pie-Crusting Safe and Effective in Balancing Cruciate-Retained Total Knee Arthroplasty? A Comparative Study

**DOI:** 10.1016/j.artd.2023.101277

**Published:** 2023-11-20

**Authors:** Mohammad mahdi Sarzaeem, Farzad Amouzadeh Omrani, Alireza Manafi Rasi, Seyed Morteza Kazemi, Alireza Mirahmadi, Mohammad Movahedinia

**Affiliations:** a Department of Orthopedic Surgery and Traumatology, Shahid Beheshti University of Medical Sciences, Tehran, Iran; b Bone, Joint and Related Tissue Research Center, Shahid Beheshti University of Medical Sciences, Tehran, Iran

**Keywords:** Arthroplasty, Replacement, Knee, Posterior cruciate ligament, Range of motion

## Abstract

**Background:**

There is still debate over whether to sacrifice the posterior cruciate ligament (PCL) during total knee arthroplasty (TKA). Several studies reported the costs and benefits of each approach regarding technical difficulties in PCL balancing and postoperation complications. In this study, we aimed to evaluate PCL needling as a safe method for balancing the cruciate-retained TKA (CR-TKA).

**Methods:**

In this comparative study, 120 patients underwent CR-TKA and were divided into 2 groups. Fifty-four patients with an acceptable PCL tightness were included in group A, and 66 cases with a tight PCL were included in group B. In group B, needle pie-crusting of the PCL was performed instead of releasing the PCL from its insertions as the standard procedure. The participants' functional outcomes, pain severity, knee range of motion, and PCL laxity were evaluated during multiple follow-ups in 3 years postoperatively.

**Results:**

The participants' age, weight, and body mass index did not differ between the 2 groups. The mean age of the patients was 69 ± 5.9 years. The functional outcomes and range of motion of the patients in the 2 groups significantly improved after the operation compared to the preoperative status, but the postoperative score was not significantly different between groups (*P* > .05). Clinical examinations were normal in all patients in both groups in all follow-up stages.

**Conclusions:**

PCL balancing is a time-consuming yet essential step for the outcome of CR-TKA and patient satisfaction. PCL needling technique shows promising results and a few complications for PCL balancing in CR-TKA.

## Introduction

Whether to retain or sacrifice the posterior cruciate ligament (PCL) in total knee arthroplasty (TKA) is still controversial, with several studies reporting different clinical outcomes about each approach [[1], [2], [3]]. PCL, the largest ligament in the human knee, is the main resistance against the posterior translocation of the tibia over the femur and plays a crucial role in knee stability during flexion [4]. Studies that are against retaining the PCL discuss technical difficulties in PCL balancing, especially in knees with significant deformity. However, retained PCL may help maintain natural knee proprioception, movements, and stability [[3], [4], [5]]. Properly balancing PCL during cruciate-retaining TKA (CR-TKA) is inevitable, as a loose PCL may cause knee instability, while a tight PCL may result in limited flexion with high pressure on or even wearing the polyethylene insert [2]. There is a paucity of evidence on a simple yet effective technique for balancing the PCL when the surgeon decides to retain it during TKA. Pie-crusting or needling has long been used to release the superficial medial collateral ligament (MCL) in balancing the gap during TKA [6].

However, there is limited experience with this technique for PCL. We hypothesize that needling the PCL in CR-TKA to overcome the tightness and balance the flexion gap results in favorable functional outcomes and knee range of motion (ROM) with few complications. The present prospective study aims to evaluate the PCL needling technique for gap balancing in CR-TKA surgery and evaluate the short-term functional outcome of this technique by the valid, reliable, and clinically sensitive Oxford Knee Scale (OKS) [[7], [8], [9], [10]].

## Material and methods

### Patients

This clinical comparative study was conducted between 2020 and 2023 in one university hospital. Initially, 132 patients with end-stage knee osteoarthritis who underwent unilateral primary TKA during the year 2019 were enrolled in the study. Twelve patients with preoperative severe deformities, including a varus angle of more than 20 or a valgus angle of more than 25 degrees, flexion contracture of more than 30 degrees, joint subluxation of more than 1 cm, or a history of intra-articular fractures, previous knee surgery, and previous PCL injury were excluded. Finally, 120 patients with an intact PCL before surgery (as macroscopically evaluated by the surgeon M.M.S. during the operation) were analyzed. Patients were divided into 2 groups based on intervention on the PCL during the surgery; patients with normal PCL tightness without any further intervention on the PCL were considered group A, and patients with a tight PCL with subsequent PCL needle pie-crusting were considered group B. The ethical committee of Shahid Beheshti University of Medical Sciences approved the study design (IR.SBMU.RETECH.REC.1399.567), and a written consent form was obtained from all the participants. All patients were operated on in one center by one senior knee surgeon (M.M.S.).

### Surgical technique

The preoperative imaging studies and required lab data were obtained. After intravenous injection of prophylactic antibiotics in the supine position, under spinal anesthesia with a high-thigh pneumatic tourniquet, a midline skin incision was made, followed by a sub-vastus medial arthrotomy. All implanted prostheses were NexGen CR Knee, the product of Zimmer Biomet, Inc., Indiana, USA. The distal femoral and proximal tibial cuts were performed based on the preoperative planning and intraoperative findings using intramedullary and extramedullary guides, respectively. The PCL was protected in all stages by a bony island around the ligament, and there were no iatrogenic injuries to the PCL. The osteophytes were removed, and a mediolaterally balanced rectangular extension gap was reached with the required medial or lateral-sided releases by using the extension-first techniques [[11], [12], [13]], then considering the femoral component thickness, the posterior femur was appropriately cut, and the spacer/alignment block was inserted.

The PCL tension was evaluated by the surgeon at 90 degrees of flexion. A tight PCL is defined by the lifting-off of the anterior lip of the tibial trial component in 90-degree flexion [14]. Fifty-four patients with an acceptable PCL tightness were included in group A, and 66 cases with a tight PCL were included in group B.

**Figure 1:**
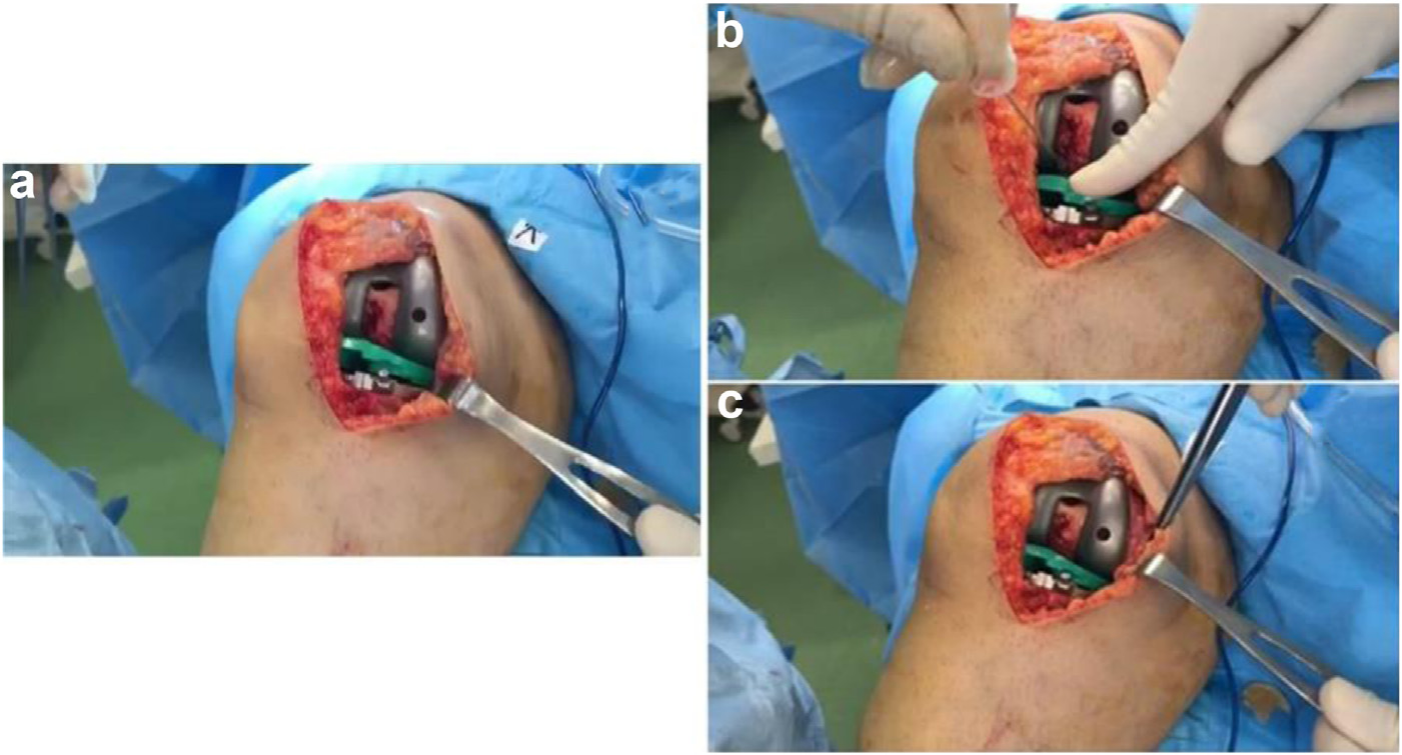
PCL tightness correction by the needling technique. (a) Positive lift-off test due to PCL tightness; (b) multiple needling of PCL; (c) correction of PCL tightness as demonstrated by negative lift-off.

Following rechecking the extension gap balance and collateral ligament tension in group B patients, another technique was used to balance the flexion gap instead of releasing the PCL from its tibial or femoral insertions as the standard procedure. This technique is based on the needle pie-crusting of the PCL while the tibial and femoral trial components are kept in place, similar to the method introduced for the MCL needling [6]. However, unlike MCL, PCL is an intra-articular ligament, which may affect the ligament healing potential of needle pie-crusting site, and this difference has been considered in final evaluations. The PCL was punctured in different sites 3 to 5 times using a 16-gauge injection needle in 90-degree flexion each time, rechecking the tightness. Then needling was continued until the following: 1. resolution of the lift-off of the anterior lip of the tibial trial component in 90 degrees flexion; 2. posterior displacement of tibia toward femur more than 1-2 millimeters; and 3. leveling of the medial femoral condyle and medial tibial plateau contact point to the posterior one-third of the tibia trial (Fig. 1). If the PCL ultimately failed due to multiple needlings, a change to PCL substituting arthroplasty was planned, although this was not the case in our patients.

After both the flexion and extension gaps were balanced with normal tension of the collateral ligaments, the femoral and tibial components were implanted using hi-fatigue cement, and the patellar tracking was evaluated. The patella did not resurface in any of our cases. The surgical wound was closed in a standard manner. Two patients in group A and one in group B had minor postoperative cellulitis on the second day but recovered without any further complications. All the other patients were discharged the following day with A.S.A. 80 mg twice daily as venous thromboembolism prophylaxis and no antibiotics. Patients were followed up at 1 month, 3 months, 6 months, 1 year, and 3 years postoperatively.

The participants' functional outcomes and pain severity were evaluated 2 weeks before and 3 years after the surgery according to OKS [15] questionnaire. The knee ROM was measured at the final follow-up. The validated version of OKS in the official language of our country was used [16].

The PCL laxity and function were clinically checked by tibial posterior sagging in knee flexion relative to the femur, the quadriceps active test [17], and the posterior drawer test at every follow-up visit of patients. These tests were performed by an orthopedic surgeon blinded to the study groups.

### Statistical analysis

In our study, the null hypothesis represents “noninferiority”, and based on ROM from a previous similar study, sample size were calculated [18]. Considering α = 5% and 1−β (power) = 95%, the calculated sample size for each group was 57.

All statistical tests were performed using SPSS software version 19 (IBM Corp. Released 2010. IBM SPSS Statistics for Windows, Version 19.0. Armonk, NY) in the form of mean, standard deviation (SD), and median. The Kolmogorov-Smirnov test was used to test the normality of distributions. Parametric variables were compared by independent t-tests between the 2 groups and by paired t-tests within the same group. Nonparametric variables were evaluated by the Mann-Whitney U test between the 2 groups and by the Wilcoxon test within the same group. Chi-square and McNemar's tests assessed differences in categorical variables between the 2 groups and within the same group, respectively. A *P*-value of less than 0.05 was set as the threshold for statistical significance.

## Theory

We believe that needle pie-crusting of the PCL during TKA to balance a tight flexion gap does not increase potential postoperative complications related to PCL insufficiency, resulting in acceptable functional outcomes.

## Results

In this study, we evaluated the functional outcomes and ROM of PCL needling in CR-TKA for balancing the flexion gap in 120 patients. Fifty-three patients underwent the operation on the right knee and 67 on the left knee. The mean age of the patients was 69 ± 5.9 years. One hundred seventeen patients were female, and 3 were male (2 males in group A and one in group B). The prosthesis survival rate was 100% at 3 years for both groups, with no periprosthetic infections or other complications.

The statistical analysis showed that the participants' age, height, weight, and body mass index (BMI) were not significantly different between the 2 groups. The tibial posterior slope during surgery was 3.1 ± 0.2 degrees in group A and 3.2 ± 0.1 degrees in group B and was not significantly different between the 2 groups.

We used the OKS to clinically evaluate the functional outcomes of patients after TKA. The OKS of the patients in the 2 groups significantly improved after the operation compared to the preoperative status, but the postoperative score was not significantly different between groups A and B (*P* = .67).

Also, there was no correlation between preoperative and postoperative OKS results and the patients' age, height, weight, and BMI. The categorization of age (≤60, 61-65, 66-70, 71-75, 76-80, >80) and BMI (≤20, >20-25, >25-30, >30) likewise failed to find any relation between OKS results and different age and BMI groups. Subjectively, all patients in both groups reported no problem with stair climbing and getting up from a chair at the end of the final follow-up.

The mean (SD) postoperative knee flexion range measured at year 3 was 135 ± 7.3 degrees for group A and 136 ± 6.5 degrees for group B. The statistical analysis didn't show any differences in knee ROM between the 2 groups (*P* = .625), although each group significantly improved compared to the preoperative status. These results are summarized in Table 1.

**Table 1:** Demographic data, Oxford knee score, and range of motion of the patients.

Variable	Needling group (mean ± SD)	Control group (mean ± SD)	Total (mean ± SD)	*P*-value
Age (years)	69.3 ± 5.7	68.7 ± 6.2	69 ± 5.9	.71
Height (centimeters)	159.5 ± 7.1	158.6 ± 7.9	159.0 ± 7.5	.66
Weight (kilograms)	77.2 ± 13.1	75.7 ± 11.2	76.4 ± 12.1	.62
BMI (kg/m^2^)	30.3 ± 4.4	30.2 ± 4.8	30.2 ± 4.5	.91
OKS before TKA	19.2 ± 3.4	19.6 ± 3.9	17.9 ± 4.9	.61
OKS after TKA	37.7 ± 6.3	38.2 ± 2.5	38 ± 4.7	.67
ROM before TKA (degrees)	101.2 ± 14	100.2 ± 13	100.75 ± 13.6	.81
ROM after TKA (degrees)	135 ± 7.3	136 ± 6.5	135.45 ± 6.9	.625

OKS, Oxford knee score; BMI, body mass index; ROM, range of motion.

The PCL laxity and function were clinically evaluated by physical examination using 3 different tests in every patient follow-up visit. The test results turned out to be normal (defined as a tibial anterior offset relative to the femur in 90-degree flexion, maximum 1+ tibial posterior translocation relative to the femur, and a negative quadriceps active test) in all patients in both groups in all stages of follow-up.

## Discussion

The main finding of this study was an acceptable functional outcome and knee ROM and PCL function following CR-TKA in cases where PCL was balanced by the needling technique. Balancing the flexion-extension gap is crucial in achieving a stable and well-functioning knee during TKA [6]. Despite the ever-improving design of knee prostheses in recent years, a notable percentage (nearly 20%-50%) of patients undergoing TKA report residual pain or functional impairment [15]. It denotes that further attempts are needed to achieve appropriate outcomes. The preservation of natural anatomical elements and their functions may be one of these attempts; hence, PCL retention during TKA might help achieve the kinematics close to the natural knee.

Balancing PCL in CR-TKA and its functional adaptation to the prosthesis is challenging. The attenuated improper PCL tension may impair the functional outcome [14]. The tight PCL may impose more stress on the polyethylene insert and cause its wear or dislocation [19]. Furthermore, it may result in a paradoxical roll-forward instead of the expected rollback of the femoral condyles [19,20].

Several methods of gap balancing in CR-TKA have been proposed with different results. After balancing the extension gap, the standard manner of tackling a tight but mediolaterally stable flexion gap is to release the PCL from its femoral or tibial attachments after removing the trial components or downsloping the tibia by recutting the proximal tibia [14]. This method is time-consuming and, in the cases of tibial downsloping, compromises the patient's bone stock. We believe that needling the PCL, like the method previously described to release MCL [6,[21], [22], [23]], is safe and effective in balancing the flexion gap in CR-TKA with normal collateral ligamentous tension.

The routine method for balancing PCL is gradually releasing it from its origins on the superior border of the posterior tibial sulcus or its femoral insertion. These techniques are challenging, not fully controllable, and may result in complete dysfunction of PCL and an inappropriate outcome of TKA. It is also time-consuming, especially in the tibial release method, because the surgeon should remove the trial component, release the PCL, set the component again, and check the balancing. This process may need to be performed multiple times.

The balancing of PCL by the needling technique could be performed without removing the trial components, so it seems to be time-saving, and the real-time effect on the flexion gap and tibial tray lift-off are easily observed each time the ligament is needled. Furthermore, it can be done in all the operation steps, even after the complete insertion of the main implant components.

There might be a concern about the integrity and function of the “needled” PCL. We did not notice any PCL-induced instability by physical examination during a 3-year follow-up, which shows the short-term preservation of the function of PCL despite needling. Also, the patients reported no problem in stair climbing and standing up from a chair, the 2 activities that need a functional PCL the most. We believe needling conserves full PCL integrity, so it rarely causes complete rupture of the PCL, as seen in our patients.

The factors that may impact patient outcomes after TKA include age, gender-antecedent diagnosis, body mass index, baseline disorders, severity, comorbidities, and psychological and socioeconomic status [15]. As indicated, confounding factors like weight, height, and, subsequently, BMI had no effect on or correlation with the outcomes of this technique.

Regarding complications, we did not encounter any significant problems in our experience with this technique, neither intraoperatively nor postoperatively.

De-Si Ma et al [18] reported acceptable outcomes following pie crusting and repairing PCL in CR-TKA on 176 patients with 5 years of follow-ups. They used a combination of pie crusting and repairing techniques, but we only used needling with comparable outcomes.

The main limitation of our study was the lack of a matched control group in a randomized controlled study design with a standard PCL release method to better evaluate and compare the results. We believe that larger comparative studies should be conducted to further evaluate this potential technique's effectiveness and safety in TKA.

## Conclusions

PCL balancing is essential in the outcome of CR-TKA. Needle pie-crusting seems to be an appropriate technique in TKA operations, which makes retained PCLs balanced without any complication. In this way, the anatomical structure of the knee remains intact with a proper prognosis. We propose the PCL needling technique as a potential substitute to the standard PCL release with promising results and few complications for PCL balancing in CR-TKA.

## Conflicts of interest

The authors declare there are no conflicts of interest.

For full disclosure statements refer to https://doi.org/10.1016/j.artd.2023.101277.

## Ethical approval and consent to participate

The institutional review board approved the study before the start of the project. Approval was granted by the Research Medical Ethics Committee of Shahid Beheshti University of Medical Sciences (IR.SBMU.RETECH.REC.1399.567). Informed consent was obtained from all the patients prior to the surgery.

## Availability of data and materials

The data that support the findings of this study are available from Shahid Beheshti University of Medical Sciences, but restrictions apply to the availability of these data, which were used under license for the current study and so are not publicly available. Data are, however, available from the corresponding author upon reasonable request and with permission of Shahid Beheshti University of Medical Sciences.

## Authors' contributions

M.M.S., F.A.O., A.M.R., S.M.K., A.M., and M.M. performed the investigation. M.M.S., F.A.O., A.M.R., S.M.K., A.M., and M.M. drafted, reviewed, and edited the paper. All authors read and approved the final manuscript.

## Appendix A Supplementary data

Conflict of Interest Statement for Omrani

Conflict of Interest Statement for Kazemi

Conflict of Interest Statement for Manafi Rasi

Conflict of Interest Statement for Mirahmadi

Conflict of Interest Statement for Movahedinia

Conflict of Interest Statement for Sarzaeem
