# Supplementary material for: Is Posterior Cruciate Ligament Needle Pie-Crusting Safe and Effective in Balancing Cruciate-Retained Total Knee Arthroplasty? A Comparative Study
Source: Arthroplast Today. 2023 Nov 20;24:101277. doi: 10.1016/j.artd.2023.101277 (PMC10696238; doi:10.1016/j.artd.2023.101277)
Supplement: Conflict of Interest Statement for Manafi Rasi [file mmc3.pdf]

CONFLICT OF INTEREST STATEMENT

American Association of Hip and Knee Surgeons

(Adopted from the American Academy of Orthopaedic Surgeons disclosure statement)

The following form **must be filled out completely and submitted by each author (example, 6 authors, 6 forms). All items require a response. If there is no relevant disclosure for a given item, enter "*None*."**

"Is Posterior Cruciate Ligament Needle Pie-Crusting Safe and Effective in Balancing Cruciate-Retained Total Knee Arthroplasty? A comparative study"

Manuscript Title

1. Royalties from a company or supplier (The following conflicts were disclosed)

   None

2. Speakers bureau/paid presentations for a company or supplier (The following conflicts were disclosed)

   None

3A. Paid employee for a company or supplier (The following conflicts were disclosed)

   None

3B. Paid consultant for a company or supplier (The following conflicts were disclosed)

   None

3C. Unpaid consultants for a company or supplier (The following conflicts were disclosed)

   None

4. Stock or stock options in a company or supplier (The following conflicts were disclosed)

   None

5. Research support from a company or supplier as a Principal Investigator (The following conflicts were disclosed)

   None

6. Other financial or material support from a company or supplier (The following conflicts were disclosed)

   None

7. Royalties, financial or material support from publishers (The following conflicts were disclosed)

   None

8. Medical/Orthopaedic publications editorial/governing board (The following conflicts were disclosed)

   None

9. Board member/committee appointments for a society (The following conflicts were disclosed)

   None

Each author must sign AND print or type his/her name, date and submit a separate form

In addition, one BLINDED Conflict of Interest form (no author names used) should be submitted per manuscript with all author disclosures.

Alireza Manafi rasi	Alireza Manafi rasi	2023-03-25
Author Name (Print or Type)	Author Signature	Date
